# Preface

**DOI:** 10.3205/cto000139

**Published:** 2016-12-15

**Authors:** Jochen A. Werner

**Affiliations:** 1 Universitätsklinikum Essen, Germany

## Preface

Dear Colleagues, 

it is a great pleasure for me to present this special issue that was written by experts of our discipline and published at the occasion of the 87th Annual Meeting of the German Society of Otolaryngology, Head and Neck Surgery. 

The annual meeting of 2016 has the heading of “Evidence and transparence in Otolaryngology – ENT, quo vadis?” which aims at defining the position of our discipline. Among the different diseases diagnosed and treated in otolaryngology, there are quite some, where therapy is not performed on the basis of the results of modern evidence-based medicine but on extensive experience. Of course, criticism is often justified when dealing with the topic of evidence-base, however, it is essential that we define the position of our discipline also in this context. With this background, I invited 9 experts of this field to analyze important areas of otolaryngology, head and neck surgery under the mentioned aspects. 

The introduction includes the definition of the terms of evidence and evidence gaps, then a detailed analysis of a meanwhile performed qualitative survey about the identification of evidence gaps in daily routine will follow. Only when we identify evidence gaps as such – and this applies to practice and clinic – we will be able to define topics that have to be dealt with in multicenter trials with high priority. Those two introductory chapters are followed by 6 articles that focus on definable evidence and evidence gaps, i.e. on non-tumorous diseases of the tonsils, nasal obstruction and rhinosinusitis, disturbed middle ear ventilation and otitis media, furthermore on the treatment of tinnitus, surgery of laryngeal carcinomas, and pharmacotherapy of non-tumorous diseases of the head and neck. This issue ends with an intensive description of the inclusion of study results in practical guidelines and their implementation in the clinic. 

I want to cordially thank all authors for their enormous efforts to create those articles and I am convinced that the present issue will turn out to be a useful source of information and provide supporting arguments in order to justify certain procedures and to initiate study projects. 

Prof. Dr. Jochen A. Werner

President of the German Society of Otolaryngology, Head and Neck Surgery

(Figure 1 (Fig. 1))

## Figures and Tables

**Figure 1:**
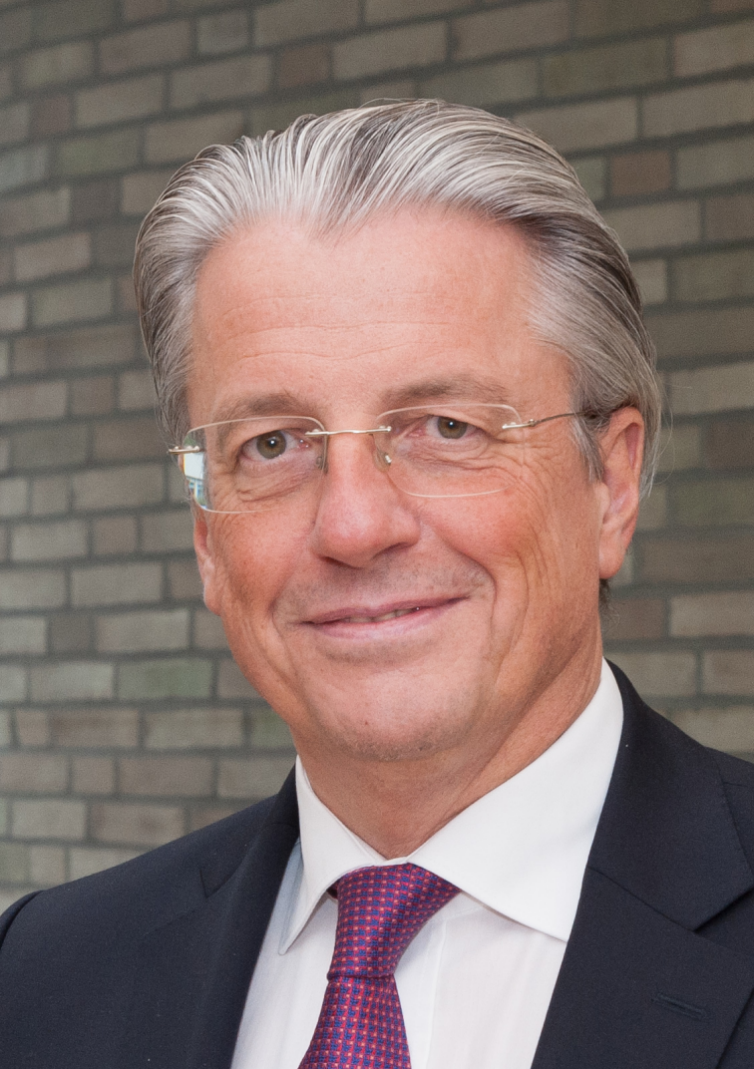
Prof. Dr. Jochen A. Werner

